# Association of methylmalonic acid with erectile dysfunction and the mediating role of endothelial activation: a population-based study of NHANES 2001-2004

**DOI:** 10.1093/sexmed/qfag054

**Published:** 2026-07-06

**Authors:** Guang-Jie Li, Chao-Hong Zhang, Li-Xing Jiang, Hai-Qing Fan, Hui-Long Fang, Yu-Ye Wu

**Affiliations:** Department of Urology, The Second Affiliated Hospital of Fujian University of Traditional Chinese Medicine, Fuzhou 350003, Fujian, China; Department of Urology, The Second Affiliated Hospital of Fujian University of Traditional Chinese Medicine, Fuzhou 350003, Fujian, China; Department of Urology, The Second Affiliated Hospital of Fujian University of Traditional Chinese Medicine, Fuzhou 350003, Fujian, China; Department of Urology, The Second Affiliated Hospital of Fujian University of Traditional Chinese Medicine, Fuzhou 350003, Fujian, China; Department of Urology, The Second Affiliated Hospital of Fujian University of Traditional Chinese Medicine, Fuzhou 350003, Fujian, China; Department of Urology, The Second Affiliated Hospital of Fujian University of Traditional Chinese Medicine, Fuzhou 350003, Fujian, China

**Keywords:** erectile dysfunction, methylmalonic acid, endothelial dysfunction, oxidative stress, mitochondria, NHANES, epidemiology, Men’s sexual health

## Abstract

**Background:**

Oxidative stress, mitochondrial dysfunction, and endothelial injury are important mechanisms in erectile dysfunction (ED). Methylmalonic acid (MMA) is related to vitamin B12 metabolism, mitochondrial dysfunction, and oxidative stress, but its relationship with ED and endothelial activation remains unclear.

**Aim:**

To investigate the associations of MMA and Endothelial Activation and Stress Index (EASIX) with ED and to evaluate whether EASIX mediates the association between MMA and ED.

**Methods:**

This cross-sectional study analyzed data from 2 National Health and Nutrition Examination Survey cycles (2001-2002 and 2003-2004; overall period, 2001-2004). A total of 3306 men aged 20 years or older were included. Survey-weighted logistic regression models were used to examine the associations of continuous MMA and EASIX with ED; exploratory categorical analyses based on data-derived cutoffs were treated as sensitivity analyses. The mediating effect of EASIX on the association between MMA and ED was assessed using the distribution-of-product method.

**Outcomes:**

The primary outcome was ED, defined using NHANES item KIQ400. The primary exposures were serum MMA and EASIX, both analyzed primarily as continuous variables.

**Results:**

Higher MMA was associated with greater odds of ED after adjustment for confounding factors (OR 1.031, 95% CI, 1.008-1.056). Higher EASIX was also associated with increased odds of ED (OR 2.284, 95% CI, 1.278-4.080). In the primary continuous-variable mediation analysis, EASIX significantly mediated the association between MMA and ED, with an indirect-effect OR of 1.0022 (95% CI, 1.0004-1.0049), and the proportion mediated was 7.18%. Exploratory categorical analyses based on restricted cubic spline-derived cutoffs showed similar directions of association.

**Clinical Implications:**

Methylmalonic acid and EASIX may serve as population-level markers associated with ED risk and may help inform future studies on metabolic-endothelial pathways in male sexual dysfunction.

**Strengths and Limitations:**

Strengths of this study include the use of a nationally representative sample and survey-weighted analyses. Limitations include the cross-sectional design, self-reported assessment of ED, and the historical nature of the NHANES cycles used.

**Conclusion:**

Higher MMA and higher EASIX were associated with increased odds of ED, and EASIX partly mediated the association in continuous-variable primary analyses. These findings should be interpreted as hypothesis-generating because of the cross-sectional design.

## Introduction

Erectile dysfunction (ED) is a prevalent disorder of the male reproductive system that significantly impairs the quality of life for both patients and their partners. ^1^ The prevalence of ED is significant, with over 150 million men worldwide reported to experience varying degrees of this condition.^2^^,^^3^ The process of achieving an erection involves multiple physiological pathways, and it is essential to accurately diagnose the specific etiological factors contributing to ED.

Oxidative stress and mitochondrial dysfunction are important pathological mechanisms of ED.^4^^,^^5^ Methylmalonic acid (MMA) is a by-product of the propionate metabolic pathway, which is considered to partially reflect vitamin B12 deficiency.^6^^,^^7^ A previous study has found that the blood vitamin B12 level of ED patients may be lower than that of healthy controls.^8^ Recent evidence indicated that MMA, as a marker of oxidative stress and mitochondrial function, is closely related to cardiovascular diseases and aging,^9^^,^^10^ and its effect may be independent of vitamin B12.^11^^,^^12^ However, no studies have investigated the relationship between MMA and ED. In addition, it is well known that oxidative stress and mitochondrial function are important causes of endothelial dysfunction.^13^^,^^14^ Nevertheless, whether MMA affects the risk of ED by mediating endothelial function remains to be investigated. Recently, Endothelial Activation and Stress Index (EASIX), a convenient index of endothelial function, has been proposed and used in several disease prognosis studies.^15^^,^^16^ The relationship between EASIX and ED, as well as whether EASIX mediates the association between MMA and ED, was still unclear.

In the present study, we investigated the associations of EASIX and MMA with ED risk and evaluated the mediating role of EASIX using data from 2 completed NHANES cycles (2001-2002 and 2003-2004; overall period, 2001-2004). These cycles were selected because they provided concurrent availability of the ED questionnaire item and the laboratory variables required to derive MMA and EASIX. Subgroup analyses were performed according to age, body mass index (BMI), and diabetes status. We tested the following refutable hypothesis: among US men in NHANES 2001-2004, are higher serum MMA levels associated with greater odds of ED, and is this association partly mediated through higher EASIX?

## Methods

### Study design and population

In this cross-sectional study, 4954 men aged ≥20 years were identified from 2 consecutive NHANES cycles (2001-2002 and 2003-2004), yielding an overall analytic period of 2001-2004. These cycles were prespecified because they contained the ED outcome variable (KIQ400) together with the laboratory measures needed for MMA assessment and EASIX calculation. NHANES is a nationally representative survey of the noninstitutionalized US population conducted by the National Center for Health Statistics using a complex multistage probability sampling design. It collects standardized data through in-person interviews, physical examinations, and laboratory assessments.^17^ The NHANES 2001-2004 protocols were approved by the NCHS Research Ethics Review Board (Continuation of Protocol #98-12), and all participants provided written informed consent. The present analysis used publicly available, deidentified data. Participants were included if they were male and aged 20 years or older. Subjects were excluded if they had prostate cancer, missed the information for ED diagnosis, missed the measurement of MMA, lactate dehydrogenase (LDH), creatinine, and platelet count, or had outlying values of MMA or EASIX.

### Potential confounding factors and definitions

Age (<45 or ≥45 years), race (non-Hispanic White, non-Hispanic Black, Mexican American, or other), educational level (less than high school, high School grad or equivalent, or more than high school), poverty-to-income ratio (<1.3, 1.3-3.5, or ≥3.6), marital status (married/Living with partner, or never married/divorced/separated/widowed), smoked at least 100 cigarettes in life (yes or no), alcohol drinking (≤2 times/week, >2 times/week, unknown), physical activity (<450 MET×min/week or ≥450 MET×min/week), complicated with diabetes or not, hypertension or not, dyslipidemia or not, cardiovascular disease (CVD) or not, body mass index (BMI <25 kg/m^2^, 25-29.9 kg/m^2^ or ≥30 kg/m^2^), C-reactive protein (CRP) (mg/dL), white blood cell (WBC) (1000 cells/uL), and serum vitamin B12 (pmol/L) were potential covariates.

Diabetes was defined as HbA1c ≥6.5% or fasting glucose ≥126 mg/dL or 2hOGTT ≥200 mg/dL, or self-reported diagnosis of diabetes (DIQ010 - Doctor told you have diabetes), use of insulin (DIQ050) or antidiabetic drugs (DIQ07) or antidiabetic drug code (358-metabolic agents-99-antidiabetic agent). Hypertension was defined as SBP ≥130 and/or DBP ≥80 mmHg, self-reported hypertension (BPQ020), use of antihypertensive medication (BPQ040A), or medication code (40-CARDIOVASCULAR AGENTS-42, 47, 48, 49, 55, 56). CVD included angina pectoris, myocardial infarction, coronary heart disease, heart failure, and stroke.

### Main and outcome variables

Methylmalonic acid and EASIX were the main exposure variables. Serum MMA was measured in NHANES laboratories using gas chromatography–mass spectrometry according to standardized CDC laboratory protocols. Endothelial Activation and Stress Index was calculated as LDH (U/L) × creatinine (mg/dL)/platelet count (10^9/L). Methylmalonic acid and EASIX were analyzed primarily as continuous variables. Restricted cubic spline (RCS) analyses were used only to explore nonlinearity and to derive data-driven cutoffs for sensitivity analyses; therefore, the MMA cutoff of 13 nmol/dL and the EASIX cutoff of 0.5 were not treated as externally validated clinical thresholds.

Erectile dysfunction was the outcome. ED was defined using the NHANES questionnaire item KIQ400: “Many men experience problems with sexual intercourse. How would you describe your ability to get and keep an erection adequate for satisfactory intercourse?” The response options were “always or almost always able,” “usually able,” “sometimes able,” and “never able.” Consistent with prior NHANES-based ED studies, participants responding “sometimes able” or “never able” were classified as having ED, whereas those responding “usually able” or “always or almost always able” were classified as not having ED.^18^^,^^19^

### Statistical analysis

Continuous variables were described as weighted means with standard errors (SEs), and weighted t tests were used for comparisons between groups. Categorical variables were presented as numbers and weighted percentages, and Rao-Scott chi-square tests were used for comparisons between groups. Missing values are presented in Supplementary Table 1. Missing data imputation was performed using the random forest method and comparisons before and after imputation are shown in Supplementary Table 2. The masked variance unit pseudo-stratum was SDMVSTRA, and the masked variance unit pseudo-primary sampling unit was SDMVPSU. For analyses combining 2 NHANES cycles (2001-2002 and 2003-2004), MEC examination weights were recalculated according to NHANES analytic guidelines to generate 4-year combined weights. All analyses incorporated sample weights, masked variance strata (SDMVSTRA), and masked variance primary sampling units (SDMVPSU) to account for the complex survey design. Weighted univariate logistic regression was used to examine potential covariates associated with ED, and backward stepwise multivariable logistic regression was used to select covariates from variables with *P* < .05. The primary association analyses modeled MMA and EASIX as continuous variables. Exploratory sensitivity analyses modeled MMA as <13 vs ≥13 nmol/dL and EASIX as <0.5 vs ≥0.5 based on RCS-derived cutoffs. The primary mediation analysis used continuous MMA and continuous EASIX. A categorical mediation analysis using MMA ≥13 vs <13 nmol/dL and EASIX ≥0.5 vs <0.5 was performed as an exploratory sensitivity analysis. The mediating effect of EASIX on the association between MMA and ED was calculated using the distribution-of-product method. The M-X and Y-X + M models were established, and the confidence intervals (CIs) of the product distributions were calculated using the RMediation package. If the CIs did not contain 0, the mediation effect was considered statistically significant, but subgroup indirect effects were interpreted cautiously when the total effect was not significant. The proportion of mediation was calculated according to the indirect effect and the total effect. Odds ratios (ORs) and 95% CIs were reported. Because this was a secondary analysis of NHANES, no clinical device was operated by the authors. Statistical analyses were conducted with R version 4.2.3 (R Foundation for Statistical Computing, Vienna, Austria), using the survey, rms, randomForest, and RMediation packages.

## Results

Comparisons of the characteristics of participants with or without ED.

**Figure 1:**
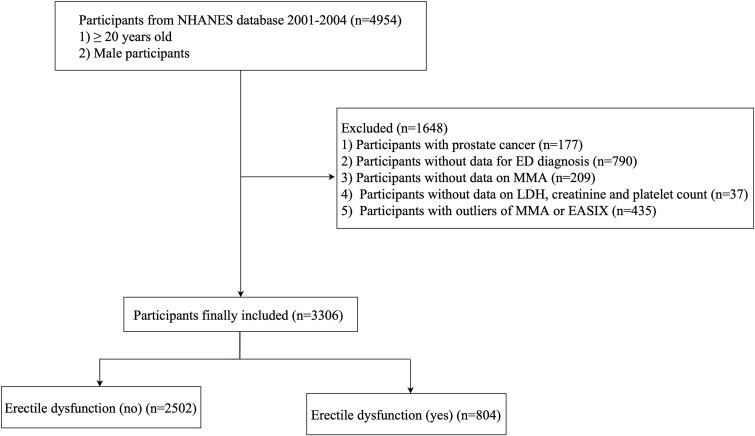
Flowchart of participant selection from 2 NHANES cycles (2001-2002 and 2003-2004; overall period, 2001-2004). Final analytic sample: no ED, *n* = 2502; ED, *n* = 804.

Among men enrolled in 2 NHANES cycles (2001-2002 and 2003-2004; overall period, 2001-2004), 4954 participants aged ≥20 years were initially identified. Participants with prostate cancer (*n* = 177), missing data on ED diagnosis (*n* = 790), missing MMA data (*n* = 209), missing LDH, creatinine, or platelet count data (*n* = 37), or outlying values of MMA or EASIX (*n* = 435) were excluded. Finally, 3306 participants were included. The screening process of participants is shown in Figure 1.

Among all participants, 804 individuals had ED. The mean EASIX was 0.51 in the overall sample, and the mean EASIX was higher in the ED group than in the non-ED group (0.54 vs 0.50). The proportion of participants with EASIX ≥0.5 was higher in the ED group than in the non-ED group (56.34% vs 44.86%). The mean MMA level was higher in the ED group than in the non-ED group (14.82 nmol/dL vs 13.39 nmol/dL). The proportion of participants with MMA ≥13 nmol/dL was higher in the ED group than in the non-ED group (61.70% vs 48.17%). Additional characteristics of the participants are presented in Table 1.

**Table 1:** Comparisons of characteristics of participants with or without ED.

		**ED**			
**Variables**	**Total (*N* = 3306)**	**No (*n* = 2502)**	**Yes (*n* = 804)**	**Statistics**	***P***
Age, *n* (%)				χ^2^ = 258.520	<.001
<45 years old	1562 (54.63)	1452 (61.92)	110 (17.62)		
≥45 years old	1744 (45.37)	1050 (38.08)	694 (82.38)		
Race, *n* (%)				χ^2^ = 0.942	.403
Non-Hispanic white	1758 (73.90)	1318 (74.03)	440 (73.28)		
Non-Hispanic black	606 (9.15)	481 (9.31)	125 (8.35)		
Mexican American	722 (8.22)	535 (8.26)	187 (8.06)		
Other	220 (8.72)	168 (8.41)	52 (10.31)		
Educational level, *n* (%)				χ^2^ = 31.619	<.001
Less than high school	889 (15.88)	575 (13.74)	314 (26.73)		
High School grad or equivalent	845 (27.97)	670 (28.39)	175 (25.85)		
More than high school	1572 (56.15)	1257 (57.87)	315 (47.42)		
Poverty-to-income ratio, *n* (%)				χ^2^ = 12.981	<.001
<1.3	759 (15.78)	538 (15.20)	221 (18.69)		
1.3-3.5	1287 (35.31)	948 (34.26)	339 (40.62)		
≥3.5	1260 (48.92)	1016 (50.54)	244 (40.70)		
Marital status, *n* (%)				χ^2^ = 23.817	<.001
Married/living with partner	2271 (69.54)	1669 (68.02)	602 (77.28)		
Never married/divorced/separated/widowed	1035 (30.46)	833 (31.98)	202 (22.72)		
Smoked at least 100 cigarettes in life, *n* (%)				χ^2^ = 58.315	<.001
No	1346 (43.17)	1107 (45.52)	239 (31.23)		
Yes	1960 (56.83)	1395 (54.48)	565 (68.77)		
Alcohol drinking status, *n* (%)				χ^2^ = 18.780	<.001
≤2 times/week	1172 (36.39)	910 (36.98)	262 (33.40)		
>2 times/week	1294 (41.30)	1050 (43.06)	244 (32.35)		
Unknown	840 (22.31)	542 (19.96)	298 (34.25)		
Physical activity, *n* (%)				χ^2^ = 8.450	.007
<450 MET×min/week	2243 (66.52)	1658 (65.27)	585 (72.85)		
≥450 MET×min/week	1063 (33.48)	844 (34.73)	219 (27.15)		
Diabetes, *n* (%)				χ^2^ = 186.798	<.001
No	2879 (90.68)	2301 (93.86)	578 (74.55)		
Yes	427 (9.32)	201 (6.14)	226 (25.45)		
Hypertension, *n* (%)				χ^2^ = 66.927	<.001
No	1453 (48.37)	1252 (52.12)	201 (29.34)		
Yes	1853 (51.63)	1250 (47.88)	603 (70.66)		
Dyslipidemia, *n* (%)				χ^2^ = 29.324	<.001
No	816 (24.63)	671 (26.30)	145 (16.15)		
Yes	2490 (75.37)	1831 (73.70)	659 (83.85)		
CVD, *n* (%)				χ^2^ = 203.989	<.001
No	2973 (92.77)	2362 (95.46)	611 (79.12)		
Yes	333 (7.23)	140 (4.54)	193 (20.88)		
BMI, *n* (%)				χ^2^ = 6.013	0.005
<25 kg/m^2^	977 (29.43)	769 (30.33)	208 (24.87)		
25-29.9 kg/m^2^	1392 (41.20)	1047 (41.45)	345 (39.98)		
≥30 kg/m^2^	937 (29.36)	686 (28.22)	251 (35.15)		
CRP, mg/L, Mean ± SE	0.32 ± 0.01	0.30 ± 0.02	0.43 ± 0.03	t = 3.958	<.001
WBC, 1000 cells/uL, Mean ± SE	7.15 ± 0.05	7.13 ± 0.06	7.28 ± 0.10	t = 1.423	.165
Serum vitamin B12, pmol/L, Mean ± SE	383.42 ± 12.25	385.55 ± 14.41	372.61 ± 6.30	t = -0.873	.390
LDH, U/L, Mean ± SE	127.33 ± 0.89	126.69 ± 0.91	130.61 ± 1.39	t = 3.039	.005
Creatinine, mg/dL, Mean ± SE	0.99 ± 0.01	0.98 ± 0.01	1.00 ± 0.01	t = 1.336	.192
Platelet count, 1000 cells/uL, Mean ± SE	257.86 ± 1.49	259.25 ± 1.43	250.81 ± 3.50	t = -2.538	.017
EASIX, Mean ± SE	0.51 ± 0.00	0.50 ± 0.00	0.54 ± 0.01	t = 4.234	<.001
EASIX, *n* (%)				χ^2^ = 11.916	.002
<0.5	1690 (53.25)	1351 (55.14)	339 (43.66)		
≥0.5	1616 (46.75)	1151 (44.86)	465 (56.34)		
MMA, nmol/dL, Mean ± SE	13.62 ± 0.15	13.39 ± 0.16	14.82 ± 0.26	t = 5.375	<.001
MMA, *n* (%)				χ^2^ = 21.652	<.001
<13 nmol/dL	1678 (49.60)	1363 (51.83)	315 (38.30)		
≥13 nmol/dL	1628 (50.40)	1139 (48.17)	489 (61.70)		

Abbreviations: BMI, body mass index; CRP, C-reactive protein; CVD, cardiovascular disease; EASIX, Endothelial Activation and Stress Index; ED, erectile dysfunction; LDH, lactate dehydrogenase; MMA, methylmalonic acid; SE, standard error; WBC, white blood cell.

### Associations of MMA or EASIX on the risk of ED

Age, educational level, poverty-to-income ratio, diabetes, and CVD were selected as covariates associated with ED. As shown in Table 2, higher MMA was associated with increased odds of ED in the crude model (OR 1.062, 95% CI, 1.039-1.086) and in the adjusted model (OR 1.031, 95% CI, 1.008-1.056). Higher EASIX was also associated with increased odds of ED in the crude model (OR 4.171, 95% CI, 2.188-7.951) and in the adjusted model (OR 2.284, 95% CI, 1.278-4.080). In exploratory categorical analyses based on RCS-derived cutoffs, participants with MMA ≥13 nmol/dL had greater odds of ED than those with MMA <13 nmol/dL (adjusted OR 1.413, 95% CI, 1.103-1.809), and participants with EASIX ≥0.5 had greater odds of ED than those with EASIX <0.5 (adjusted OR 1.395, 95% CI, 1.070-1.820).

**Table 2:** Associations of continuous MMA and EASIX with the risk of ED, with exploratory categorical sensitivity analyses.

	**Model 1**		**Model 2**	
**Variables**	**OR (95% CI)**	***P***	**OR (95% CI)**	***P***
MMA	1.062 (1.039-1.086)	<.001	1.031 (1.008-1.056)	.011
MMA				
<13 nmol/dL	Ref		Ref	
≥13 nmol/dL	1.733 (1.359-2.211)	<.001	1.413 (1.103-1.809)	.008
EASIX	4.171 (2.188-7.951)	<.001	2.284 (1.278-4.080)	.007
EASIX				
<0.5	Ref		Ref	
≥0.5	1.587 (1.205-2.088)	.002	1.395 (1.070-1.820)	.016

Abbreviations: CI, confidence interval; EASIX, Endothelial Activation and Stress Index; MMA, methylmalonic acid; OR, odds ratio.

### Mediating effect of EASIX on the relationship between MMA and ED

**Figure 2:**
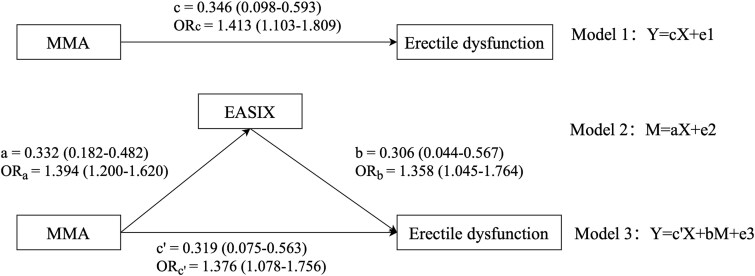
Mediation model illustrating the role of Endothelial Activation and Stress Index (EASIX) in the association between methylmalonic acid (MMA) and erectile dysfunction (ED). Path c represents the total effect of MMA on ED, path a the effect of MMA on EASIX, path b the effect of EASIX on ED, and path c’ the direct effect of MMA on ED after accounting for EASIX. The adjusted model was controlled for age, educational level, poverty-to-income ratio, diabetes, and cardiovascular disease.

As shown in Table 3, the primary continuous-variable mediation analysis showed that EASIX significantly mediated the relationship between MMA and ED. The adjusted indirect-effect OR was 1.0022 (95% CI, 1.0004-1.0049), and the product-distribution beta was 0.0022 (95% CI, 0.0003-0.0049), with a mediation proportion of 7.18% (Figure 2). In the exploratory categorical mediation analysis, the adjusted indirect-effect OR was 1.107 (95% CI, 1.018-1.229), representing the indirect effect of MMA ≥13 nmol/dL relative to MMA <13 nmol/dL operating through EASIX ≥0.5 (Supplementary Table 3).

**Table 3:** Primary continuous-variable mediation analysis of EASIX in the association between MMA and ED.

**Variables**	**Model 1**	**Model 2**
Total effect, OR (95% CI)	1.062 (1.039-1.086)	1.031 (1.008-1.056)
Direct effect, OR (95% CI)	1.057 (1.034-1.081)	1.029 (1.005-1.053)
Product distribution, β (95% CI)	0.005 (0.002-0.009)	0.0022 (0.0003-0.0049)
Indirect effect, OR (95% CI)	1.005 (1.002-1.009)	1.0022 (1.0004-1.0049)
Proportion of mediation, %	8.31	7.18

Abbreviations: ED, erectile dysfunction; MMA, methylmalonic acid; OR, odds ratio.

### Subgroup analysis on the mediating effect of EASIX on the relationship between MMA and ED

Subgroup mediation analyses are presented in Supplementary Tables 4 and 5. In continuous-variable analyses, indirect effects were statistically significant among participants aged ≥45 years, participants with BMI ≥25 kg/m2, and participants without diabetes, with mediation proportions of 11.00%, 13.50%, and 5.77%, respectively. In the diabetes subgroup, the total effect of MMA on ED was not statistically significant in either the categorical analysis (OR 1.437, 95% CI, 0.700-2.949) or the continuous analysis (OR 1.030, 95% CI, 0.969-1.095); therefore, the indirect-effect estimates in this subgroup were interpreted as exploratory and hypothesis-generating rather than conclusive evidence of mediation.

## Discussion

To our knowledge, this is the first population-based study to explore the potential mediating role of endothelial activation in the association between MMA and ED. In this study, higher continuous MMA and higher continuous EASIX were associated with increased odds of ED, and EASIX partly mediated the MMA-ED association in the primary continuous-variable mediation analysis. Exploratory categorical analyses based on data-derived cutoffs showed similar directions of association. These findings suggest that EASIX and MMA may represent candidate biomarkers associated with ED risk. ED has also been recognized as an early symptom and risk factor for vascular and peripheral diseases, and recent single-cell transcriptomic analysis highlights mitochondrial dysfunction as a key contributor to ED pathogenesis.^20^

Previously, the antioxidant defense profile of testicular mitochondria exhibits age-related modifications that may play a crucial role in regulating physiological functions, and mitochondrial dysfunction is negatively correlated with and may affect, several reproductive function parameters.^21^ Endothelial dysfunction in corpus cavernosum tissue is associated with reduced nitric oxide production and impaired nitric oxide activity, potentially leading to ED.^22^^,^^23^ The cyclic guanosine monophosphate pathway is an important component of penile erection and is positively correlated with nitric oxide production.^24^ Experimental studies have also shown that chronic prostatitis/chronic pelvic pain syndrome and chronic sleep deprivation can impair erectile function through oxidative stress, endothelial dysfunction, apoptosis, and corporal fibrosis.^25^^,^^26^ Additional recent mechanistic work in diabetic ED models supports the importance of oxidative stress, microvascular injury, and endothelial nitric oxide signaling: inhibition of inducible nitric oxide synthase alleviated oxidative stress and microvascular injury in diabetic rats with ED,^27^ and human umbilical cord mesenchymal stem cell treatment restored erectile function in diabetic rats by inhibiting TLR4 and increasing VEGF and eNOS expression.^28^ These findings place the present epidemiologic results in a broader experimental context in which oxidative stress and endothelial dysfunction are plausible contributors to ED.

Methylmalonic acid is a water-soluble organic acid that has gained recognition as a reliable functional marker for identifying vitamin B12 deficiency.^29^ Vitamin B12 serves as an essential cofactor for L-methylmalonyl-CoA mutase, facilitating the conversion of methylmalonyl-CoA into succinyl-CoA.^30^ In cases of vitamin B12 deficiency, the enzymatic activity of L-methylmalonyl-CoA mutase is compromised, leading to the accumulation of methylmalonyl-CoA and its subsequent conversion into MMA.^31^ Methylmalonic acid is also considered a marker of mitochondrial dysfunction and has been reported to be related to the risk of prostate cancer.^32^^,^^33^ Endothelial Activation and Stress Index is a composite index based on LDH, creatinine, and platelet count; these components are indirect surrogate markers rather than direct endothelial-function measurements.^34^ Direct evidence for a specific high-MMA-to-EASIX-to-ED pathway remains limited. Therefore, the mechanistic hypothesis in the present study is based on the broader chain linking MMA to mitochondrial dysfunction and oxidative stress,^7^^,^^35^ and linking oxidative stress and mitochondrial dysfunction to endothelial dysfunction.^13^^,^^14^ In the present study, higher EASIX and MMA levels were associated with increased odds of ED, and EASIX partly mediated the association between MMA and ED. Furthermore, a recent population-based analysis found that a higher oxidative balance score was associated with a decreased risk of ED and underscored that ED may precede cardiovascular, neurodegenerative, and other systemic diseases.^36^ Another NHANES-based study reported that a magnesium deficiency score was positively associated with ED risk and proposed that magnesium deficiency may impair erectile function through reduced nitric oxide production and increased oxidative stress.^37^

The samples in this study were obtained from the NHANES database through multistage complex sampling and weighted processing and are representative of the US civilian noninstitutionalized population. There were some limitations. First, the analysis was based on NHANES 2001-2004, which may reduce the contemporary relevance of the findings. However, these cycles were selected because they offered concurrent availability of the ED questionnaire variable and the laboratory measures required for MMA and EASIX assessment. Therefore, the present findings should be interpreted as hypothesis-generating and require validation in more contemporary cohorts. Second, this study was cross-sectional, and causal associations between MMA, EASIX, and ED cannot be established. Mediation estimates in a cross-sectional analysis should be interpreted as statistical decomposition rather than proof of temporal causality. Third, the MMA and EASIX cutoffs were derived from the analytic dataset using RCS and were not externally validated clinical thresholds; for this reason, categorical analyses were repositioned as exploratory sensitivity analyses, and the continuous-variable models were emphasized as the primary analyses. Fourth, ED was assessed with a single NHANES questionnaire item rather than a validated multidomain instrument such as the International Index of Erectile Function. Although classifying “sometimes able” and “never able” as ED is consistent with prior NHANES-based studies,^18^^,^^19^ this approach may capture a broader spectrum of erectile difficulty than studies defining ED only as “never able,” and misclassification is possible. Fifth, EASIX is an indirect composite marker and does not directly measure endothelial nitric oxide bioavailability or penile endothelial function. Sixth, in the diabetes subgroup, the total effect of MMA on ED was not statistically significant in either categorical or continuous analyses, so any indirect-effect signal in this subgroup should be regarded as exploratory and requiring validation in larger prospective studies. Finally, although demographic, comorbidity, and lifestyle factors were considered, some potential confounding factors, such as treatment information for ED, were not included in the analysis because of limitations of the NHANES database.

## Conclusion

In this population-based study of NHANES 2001-2004, higher MMA and higher EASIX were associated with increased odds of ED, and EASIX partly mediated the association between MMA and ED in the primary continuous-variable analysis. These findings provide hypothesis-generating epidemiological evidence supporting a potential metabolic-endothelial pathway in ED.

## Supplementary Material

SM-26-0417_Supplementary_Tables_1-5_qfag054

## Statement of agree publication

All authors have read this manuscript and agree to publish this manuscript.
