# Supplementary material for: Association of methylmalonic acid with erectile dysfunction and the mediating role of endothelial activation: a population-based study of NHANES 2001-2004
Source: Sex Med. 2026 Jul 6;14(5):qfag054. doi: 10.1093/sexmed/qfag054 (PMC13336635; doi:10.1093/sexmed/qfag054)
Supplement: SM-26-0417_Supplementary_Tables_1-5_qfag054 [file sm-26-0417_supplementary_tables_1-5_qfag054.docx]

Supplementary Tables

**Supplementary Table 1. The status of missing values**

BMI: body mass index.

**Supplementary Table 2. Comparisons of data before and after missing value imputation**

S.E: standard error; t: weighted t test; chi-square: Rao-Scott chi-square test; BMI: body mass index.

**Supplementary Table 3. Exploratory categorical mediation analysis of EASIX in the association between MMA and ED**

MMA was modeled as ≥13 versus <13 nmol/dL and EASIX as ≥0.5 versus <0.5. OR: odds ratio; CI: confidence interval. Model 1: weighted univariate logistic regression model. Model 2: multivariable logistic regression model adjusted for age, educational level, poverty-to-income ratio, diabetes, and CVD.

**Supplementary Table 4. Exploratory subgroup mediation analysis using categorical MMA and EASIX**

MMA was modeled as ≥13 versus <13 nmol/dL and EASIX as ≥0.5 versus <0.5. Indirect-effect estimates are exploratory when the corresponding total effect is not statistically significant.

**Supplementary Table 5. Subgroup mediation analysis using continuous MMA and EASIX**

MMA and EASIX were modeled as continuous variables. Indirect-effect estimates are exploratory when the corresponding total effect is not statistically significant.

| Variables | n **(**%) |
| --- | --- |
| Educational level | 2 **(**0.06) |
| Marital status | 2 **(**0.06) |
| Smoked at least 100 cigarettes in life | 2 **(**0.06) |
| Serum vitamin B12 | 4 **(**0.12) |
| BMI | 60 **(**1.81) |
| Poverty-to-income ratio | 174 **(**5.26) |

| Variables | Before imputation | After imputation | Statistics | *P* |
| --- | --- | --- | --- | --- |
| Educational level, n **(**%) |  |  | χ² = 1.434 | 0.241 |
| Less than high school | 889 **(**15.89) | 889 **(**15.88) |  |  |
| High School grad or equivalent | 845 **(**27.99) | 845 **(**27.97) |  |  |
| More than high school | 1570 **(**56.12) | 1572 **(**56.15) |  |  |
| Marital status, n **(**%) |  |  | χ² = 1.950 | 0.173 |
| Married / Living with partner | 2269 **(**69.50) | 2271 **(**69.54) |  |  |
| Never married / Divorced / Separated / Widowed | 1035 **(**30.50) | 1035 **(**30.46) |  |  |
| Smoked at least 100 cigarettes in life, n **(**%) |  |  | χ² = 1.531 | 0.226 |
| No | 1346 **(**43.18) | 1346 **(**43.17) |  |  |
| Yes | 1958 **(**56.82) | 1960 **(**56.83) |  |  |
| Serum vitamin B12, pmol/L, Mean ± S.E | 383.49 ± 12.26 | 383.42 ± 12.25 | t = -1.078 | 0.290 |
| BMI, kg/m^2^, Mean ± S.E | 28.09 ± 0.11 | 28.09 ± 0.11 | t = -0.362 | 0.720 |
| Poverty-to-income ratio, Mean ± S.E | 3.25 ± 0.05 | 3.25 ± 0.04 | t = 0.184 | 0.855 |

| Variables | Model 1 | Model 2 |
| --- | --- | --- |
| Total effect, OR (95% CI) | 1.733 (1.359-2.211) | 1.413 (1.103-1.809) |
| Direct effect, OR (95% CI) | 1.669 (1.317-2.116) | 1.376 (1.078-1.756) |
| Product distribution, β (95% CI) | 0.164 (0.057-0.294) | 0.102 (0.018-0.206) |
| Indirect effect, OR (95% CI) | 1.179 (1.059-1.342) | 1.107 (1.018-1.229) |
| Proportion of mediation, % | 29.88 | 29.36 |

| Subgroups (ED/total) | Total effect  OR (95% CI) | Direct effect  OR (95% CI) | Indirect effect  OR (95% CI) | Proportion of mediation |
| --- | --- | --- | --- | --- |
| Age |  |  |  |  |
| <45 (n=110/1562) | 0.723 (0.450-1.161) | 0.754 (0.468-1.215) | 0.842 (0.660-0.997) |  |
| ≥45 (n=694/1744) | 1.761 (1.366-2.270) | 1.687 (1.311-2.171) | 1.212 (1.049-1.470) | 33.94 |
| BMI |  |  |  |  |
| <25 (n=208/977) | 1.352 (0.840-2.176) | 1.315 (0.811-2.133) | 1.089 (0.981-1.293) |  |
| ≥25 (n=596/2329) | 1.426 (1.039-1.958) | 1.387 (1.009-1.908) | 1.119 (1.001-1.290) | 31.70 |
| Diabetes |  |  |  |  |
| No (n=578/2879) | 1.399 (1.061-1.844) | 1.378 (1.047-1.814) | 1.066 (0.997-1.170) | 19.07 |
| Yes (n=226/427) | 1.437 (0.700-2.949) | 1.263 (0.635-2.511) | 1.842 (1.029-4.223) |  |

| Subgroups | Total effect  OR (95% CI) | Direct effect  OR (95% CI) | Indirect effect  OR (95% CI) | Proportion of mediation  OR (95% CI) |
| --- | --- | --- | --- | --- |
| Age |  |  |  |  |
| <45 (n=110/1562) | 0.976 (0.926-1.028) | 0.980 (0.931-1.032) | 0.995 (0.989-0.999) |  |
| ≥45 (n=694/1744) | 1.049 (1.023-1.075) | 1.044 (1.018-1.071) | 1.005 (1.002-1.009) | 11.00 |
| BMI |  |  |  |  |
| <25 (n=208/977) | 1.034 (0.978-1.093) | 1.034 (0.978-1.093) | 1.000 (0.996-1.003) |  |
| ≥25 (n=596/2329) | 1.032 (1.005-1.059) | 1.028 (1.001-1.056) | 1.004 (1.001-1.008) | 13.50 |
| Diabetes |  |  |  |  |
| No (n=578/2879) | 1.030 (1.004-1.057) | 1.028 (1.002-1.055) | 1.002 (1.000-1.005) | 5.77 |
| Yes (n=226/427) | 1.030 (0.969-1.095) | 1.022 (0.963-1.084) | 1.010 (1.000-1.024) |  |
